# Publisher note: suspending submissions to *Bioengineered*

**DOI:** 10.1080/21655979.2025.2525650

**Published:** 2025-07-02

We have taken the difficult decision to suspend new submissions to *Bioengineered*. This pause will enable the Editors, Editorial Board and Taylor & Francis staff to focus fully on ongoing investigations into a large number of historical articles suspected of being the product of paper mill or systematic manipulation activity. Authors of recently submitted articles will be given the option to transfer their manuscript to an alternative Taylor & Francis journal for consideration.

As we have previously outlined^1^ ^1^Dealing with the perils of “paper mills”: How the *Bioengineered* journal fights fake science https://insights.taylorandfrancis.com/research-impact/dealing-paper-mills-bioengineered, the editorial integrity of *Bioengineered* was compromised between 2021 and 2022. There are also papers we are investigating that fall outside that period. A significant spike in submissions and requests to change the authorship of papers after submission indicate that the journal may have been the victim of a targeted paper mill.

To date, our robust response to this serious issue has included:

- Introducing a rigorous screening system for all new submissions. A specially trained in-house Content Integrity Screening team carries out a range of compliance and integrity checks, including author and data validation.
- Refreshing the journal’s editorial leadership and Board, ensuring all members are committed to acting in accordance with the Taylor & Francis Editor Code of Conduct and Reviewer Guidelines.
- Appointing an in-house Executive Editor to oversee journal and editorial performance.
- Initiating an extensive program of investigations into published articles of concern, expanding the team to resource the high number of cases.
- Retracting many compromised articles, a process which began in early 2022.

This activity has enabled us to proactively detect and prevent the publication of a sizeable amount of further paper mill content submitted to the journal.

However, there remains a substantial number of published articles of concern. We have recently introduced new functionality to Taylor & Francis Online to alert readers when an article is being investigated by our Publishing Ethics & Research Integrity team. Over 1,000 *Bioengineered* articles currently display this “under investigation” notice.

Each investigation is being carried out in accordance with our editorial policies, in alignment with the Committee on Publication Ethics principles (COPE). This is necessarily a detailed and time-consuming process, which can involve gathering and analyzing evidence, consulting experts, and giving authors appropriate opportunity to respond to any concerns.

Taylor & Francis and the *Bioengineered* editorial team are committed to fully correcting the scholarly record. Suspending submissions will enable everyone involved to support the current ethics investigations. The pause will also give us the opportunity to consider the journal’s future.

We are grateful to the current *Bioengineered* Editors and Board for their dedication to upholding the highest standards of publishing ethics.

